# Acceptability of Continuous Glucose Monitoring in Free-Living Healthy Individuals: Implications for the Use of Wearable Biosensors in Diet and Physical Activity Research

**DOI:** 10.2196/11181

**Published:** 2018-10-24

**Authors:** Yue Liao, Susan Schembre

**Affiliations:** 1 Department of Behavioral Science University of Texas MD Anderson Cancer Center Houston, TX United States; 2 Department of Family and Community Medicine College of Medicine-Tucson University of Arizona Tucson, AZ United States

**Keywords:** wearable sensors, remote monitoring, physiological monitoring, accelerometry, user experience

## Abstract

**Background:**

Wearable sensors have been increasingly used in behavioral research for real-time assessment and intervention purposes. The rapid advancement of biomedical technology typically used in clinical settings has made wearable sensors more accessible to a wider population. Yet the acceptability of this technology for nonclinical purposes has not been examined.

**Objective:**

The aim was to assess the acceptability of wearing a continuous glucose monitor (CGM) device among a sample of nondiabetic individuals, and to compare the acceptability of a CGM between a mobile diet tracking app (MyFitnessPal) and an accelerometer.

**Methods:**

A total of 30 nondiabetic adults went through a 7-day observational study. They wore a CGM sensor, tracked their diet and physical activity using the CGM receiver and MyFitnessPal, and wore an accelerometer on their waist. After the monitoring period, they completed a 10-item survey regarding acceptability of each of the study tools. Two-tailed paired-sample *t* tests were conducted to examine whether the summary acceptability scores were comparable between the CGM sensor/receiver and MyFitnessPal/accelerometer.

**Results:**

More than 90% of the study participants agreed that the CGM sensor and receiver were easy to use (28/30 and 27/30, respectively), useful (28/30 and 29/30, respectively), and provided relevant information that was of interest to them (27/30 and 28/30, respectively). The summary acceptability scores (out of a 5-point Likert scale) were mean 4.06 (SD 0.55) for the CGM sensor, mean 4.05 (SD 0.58) for the CGM receiver, mean 4.10 (SD 0.68) for MyFitnessPal, and mean 3.73 (SD 0.76) for the accelerometer.

**Conclusions:**

The high acceptability of using a CGM from this study suggests a great potential for using CGMs in nondiabetic adults in research settings. Although potential selection bias might contribute to the high acceptability in this study, the continued advancements in wearable sensor technology will make the barriers to tracking and collecting personal physiological data more and more minimal.

## Introduction

Diet and physical activity are the two leading modifiable lifestyle behaviors that could significantly impact future health outcomes such as obesity, hypertension, diabetes, and heart disease [1]. Nevertheless, in the United States, adherence to meeting the dietary and physical activity guidelines has remained low for the past few decades [2-4]. Numerous efforts have been devoted to understanding the determinants and correlates of diet and physical activity behavior with the goal of developing novel and more effective interventions to promote and sustain positive health behavior changes. In recent years, behavioral research has seen a sharp increase in the use of mobile and wearable technology in diet and physical activity assessment and interventions [5]. Some of the most widely used technologies in behavioral research include mobile apps and wearable activity trackers [5-7], reflecting researchers’ interests in utilizing wearable devices to understand and improve behavioral health.

New technologies are being developed to capture an extraordinary array of health-related information. Biosensors, wearable devices that either continuously or frequently measure physiological parameters [8,9], are becoming more affordable and accessible, providing opportunities for their application beyond clinical settings. One example of wearable biosensors with the potential to be used in behavioral research is the continuous glucose monitor (CGM). The CGM measures the concentration of glucose subcutaneously (interstitial fluid) in real time through a tiny sensor inserted under the skin [10]. It has been primarily utilized by type 1 diabetic patients treated by intensive insulin therapy to make treatment decisions that promote glycemic control [11]. In recent years, the use of CGMs has increased in primary care of patients with uncontrolled type 2 diabetes to improve patient’s self-management skills (ie, treatment adherence, lifestyle changes) [12], demonstrating a broader application potential for CGMs.

Related to the use of CGMs for disease management, an increasing number of studies have begun to use CGMs in research to examine the acute effect of dietary intake and physical activity on insulin concentrations and glucose metabolism in both diabetic [13-16] and nondiabetic populations [17-21]. For example, using CGM in free-living settings, Brynes and colleagues [13] demonstrated the beneficial effect of a low glycemic diet on the 24-hour glucose profile in type 2 diabetic individuals, as well as in healthy young people [17]. In a controlled laboratory setting (whole-room calorimeter) with 2-day CGM assessment, DiPietro and colleagues [18] found that both sustained (45 minutes) morning walking and short (15 minutes) postmeal walking improved the 24-hour glucose profile in inactive older adults without diabetes. Multiple behavioral theories (eg, self-determination theory, social cognitive theory) address the importance of perceived benefits and outcome expectancy in influencing changes in dietary and physical activity behaviors [22-24]. Since glucose is a biological marker that could be acutely impacted by diet and physical activity, the richness of data collected from a CGM could potentially be used in health behavior interventions in a way to illustrate the immediate physiological consequences of one’s behavior and subsequently encourage behavioral changes (eg, biologically based behavioral feedback).

Despite the growing utilization of CGMs in diet and physical activity research beyond the diabetic population, and its potential as a tool to promote diet and physical activity behavior change, questions have remained about the acceptability of CGMs to nondiabetic individuals. As such, the goal of this study was to describe the acceptability of a CGM and compare it to other widely used mobile diet and physical activity data collection methods (ie, MyFitnessPal and an accelerometer) from a sample of nondiabetic individuals. Knowledge gained from these results intends to support the use of CGMs in diet and physical activity research and could inform the planning and development of future diet and physical activity studies that use CGMs.

## Methods

### Study Overview

Data used for this study were from Project SENSE, an observational study aimed at testing the feasibility and utility of CGMs to detect and characterize consummatory (eating and drinking) events in free-living adults without diabetes. All study participants gave their written informed consent. Project SENSE was approved by the Institutional Review Board at the University of Texas MD Anderson Cancer Center.

### Participants

Adults were recruited through public and private announcements (eg, email listserv, word-of-mouth) around the Texas Medical Center in Houston, Texas. These included healthy individuals working/living in the communities near the medical center, as well as patients from the MD Anderson Cancer Center who were free from cancer and diabetes. Interested individuals contacted the study team to assess their eligibility to participate in the study. Eligible individuals were between ages 21 and 65 years; able to speak, read, and write in English; and had a mobile phone with internet access. Individuals were excluded if they reported being diagnosed with diabetes, reported use of any medication known to affect glucose levels (eg, corticosteroids, antidepressants, metformin), had fasting blood glucose >125 mg/dL as measured by glucometer, were pregnant or lactating, had a reported diagnosis of a chronic condition with dietary restrictions or an eating disorder, or were unable or unwilling to use a CGM. The recruitment goal was to enroll 30 study participants. This sample size was chosen based on previous mHealth studies that tested acceptability [25,26].

### Procedures

Interested individuals, who passed the initial eligibility screening, were invited for an in-person visit to have their fasting blood glucose measured by a commercially available glucometer to confirm their eligibility and enroll in the study. On enrollment, participants were introduced to the study equipment, which included a CGM system (Dexcom G4 Platinum, San Diego, CA, USA), a glucometer for CGM calibration, a mobile app to track dietary intake (MyFitnessPal), and an accelerometer to measure physical activity. The 7-day self-monitoring period started after the completion of the in-person visit. Participants were asked to track all dietary intake and exercise events using both the CGM receiver and the MyFitnessPal app. Participants were told not to change their usual behaviors during the monitoring period. Participants came back for another in-person visit on day 8 to return the study equipment and to complete an exit survey. Participants received a compensation (up to US $200) for completing the study.

### Study Tools

#### Continuous Glucose Monitor

**Figure 1:**
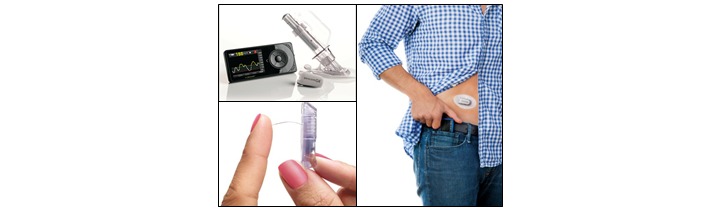
Dexcom G4 Platinum continuous glucose monitoring system.

The Dexcom G4 Platinum CGM system included a sensor, transmitter, and receiver (Figure 1). On insertion and activation of the sensor, glucose levels were recorded every 5 minutes. The receiver screen displayed the real-time glucose reading, a trend arrow indicating rate of change, and a graph showing the glucose trend in the past 24 hours. In addition, the receiver had the function to mark (ie, time stamp) eating events and exercise sessions. Participants were asked to record all eating and drinking of calorie-containing beverages using the receiver within 5 minutes of initiating the consummatory event. To ensure proper data transmission between the CGM transmitter and receiver, participants were asked to keep the receiver within 18 feet of them at all times. Lastly, with the Dexcom G4 Platinum CGM system, participants were required to perform a finger prick calibration using the supplied glucometer set at least every 12 hours.

#### Diet Tracking App

Participants kept a detailed food log (ie, food and caloric-beverage consumed, portion size, time of consumption) using the MyFitnessPal mobile app. In addition, participants were asked to take a time-stamped photo of all food and caloric beverages consumed using their mobile phone and email the images to the study coordinator at the end of each day. Food photos were used to confirm the time stamp marked in the CGM receiver for each of the recorded consummatory events. The MyFitnessPal app was also used by participants to enter all exercise events (ie, time, duration, and type).

#### Accelerometer

The ActiGraph GT3X was used to objectively measure physical activity. Participants wore the accelerometer on their waist during all waking hours, except when bathing or swimming.

### Measures

#### Acceptability

A 10-item survey was developed to assess the acceptability of all study tools used in Project SENSE. The survey items were chosen from previous mHealth feasibility studies [25,26] with a focus of addressing barriers and facilitators in the use of mHealth tools [27-29], such as convenience, value, and relevance. The survey was pilot-tested in the targeted population before launching. The response options were on a 5-point Likert scale, ranging from “strongly disagree” to “strongly agree.” All participants completed the survey during their exit visit. Two additional questions were later added to the survey to specifically ask about participants’ opinions on using a CGM for health and wellness purposes: “How likely or willing are you to use a wearable glucose sensor, like the one you used in this study, to help you achieve your health and wellness goals (healthy eating or weight management)?” and “How likely or willing would you be to use a wearable glucose sensor to help you achieve your health and wellness goals (healthy eating or weight management), if the sensor did not have to be inserted under your skin (ie, noninvasive)?” The response options were on a 5-point Likert scale, ranging from “very unlikely” to “very likely.” Half of the participants answered these two questions.

### Statistical Analysis

Statistical analyses were conducted using the SPSS version 24.0 (IBM Corp, Armonk, NY, USA). Descriptive statistics were generated for all variables, including the mean and standard deviation for continuous variables and percentages for categorical variables. Cronbach alpha for the acceptability scale ranged from .757 (CGM sensor) to .853 (MyFitnessPal). A summary score of acceptability was created for each study tool (ie, CGM sensor, CGM receiver, MyFitnessPal, and accelerometer) by calculating the mean of the 10 survey items (the Privacy item was reverse-coded). Two-tailed paired-sample *t* tests were used to examine whether the acceptability score was comparable between CGM sensor, CGM receiver, MyFitnessPal, and accelerometer. A *P* value of .05 or less was considered significant.

## Results

### Participant Characteristics

A total of 66 individuals completed the eligibility screening. Eight were ineligible due to having been diagnosed with diabetes (type I or II), four were taking medication that would impact glucose levels, two had dietary intake restrictions due to health conditions, one was unwilling to have the sensor inserted, and nine were due to other reasons (eg, age, time commitment, pregnancy, other health reasons). A total of 42 individuals, who passed the initial eligibility screening, were scheduled for an in-person visit to determine final eligibility. Of these individuals, eight did not attend their appointment and were unable to be rescheduled and four had elevated fasting blood glucose levels.

A total of 30 participants enrolled in Project SENSE. Average age was 38 (SD 13, range 24-64) years. In all, 73% (22/30) of the participants were female, 17% (5/30) were Hispanic, and 64% (19/30) were overweight or obese. Table 1 shows the detailed demographic characteristics of the participants.

Of the 30 participants, three experienced an unexpected failure of their CGM adhesive, which caused the sensor to be removed prematurely (two participants had four days’ wear and one participant had five days’ wear). All other participants wore the CGM for the entire 7-day observational period. The daily eating events recorded by participants was mean 5.6 (SD 2.2) for the CGM receiver and mean 5.4 (SD 2.1) for MyFitnessPal.

### Acceptability

Overall, more than 90% (27/30) of participants agreed with the statements regarding usability, value, relevance, and confidence for both the CGM sensor and the CGM receiver. Table 2 shows the results from the acceptability survey for all tools used in Project SENSE. Of the 15 participants who answered the additional questions regarding using CGM for health and wellness purposes, 6 (40%) indicated that they were likely to do so using a similar CGM system, and 12 (80%) indicated that they were likely to do so if the CGM system became noninvasive.

**Table 1:** Participant characteristics (N=30).

Characteristic	Participants
Age (years), mean (SD)	37.9 (13.2)
Female, n (%)	22 (73)
Hispanic, n (%)	5 (17)
Overweight, n (%)	14 (47)
Obese, n (%)	5 (17)
College graduated, n (%)	28 (93)
Full-time employed, n (%)	22 (73)

**Table 2:** Participant experiences of each study tool (N=30)^a^.

Acceptability survey item	CGM^b^ sensor, n (%)	CGM receiver, n (%)	MyFitnessPal, n (%)	Accelerometer, n (%)
Usability: this tool is easy to use and user friendly	28 (93)	27 (90)	28 (93)	28 (93)
Convenience: this tool is convenient for me to use in my everyday lives	22 (73)	19 (63)	22 (73)	18 (60)
Value: this tool is useful and beneficial	28 (93)	29 (97)	28 (93)	23 (77)
Relevance: this tool provides information that is of interest to me	27 (90)	28 (93)	27 (90)	13 (43)
Motivating: I am motivated to use this tool to track my daily behaviors	19 (63)	20 (67)	24 (80)	11 (37)
Tech support: there is adequate availability and quality of professional assistance throughout use of this tool	21 (70)	20 (67)	21 (70)	18 (60)
Confidence: I feel confident that I use this tool correctly	29 (97)	28 (93)	27 (90)	28 (93)
Privacy: I am concerned about my privacy when using this tool	1 (3)	1 (3)	4 (13)	1 (3)
Recommend: I would recommend this tool to my friends and family	19 (63)	20 (67)	28 (93)	14 (47)
Like: I like using this tool	20 (67)	23 (77)	24 (80)	17 (57)

^a^Values are number of participants who agreed or strongly agreed with the statement.

^b^Continuous glucose monitor.

**Table 3:** Mean of acceptability score for each data collection tool and comparisons of their mean scores^a^.

Data collection tool	Acceptability score, mean (SD)	Tool, absolute mean difference (SD)		
		CGM^b^ sensor	CGM receiver	MyFitnessPal
CGM sensor	4.06 (0.55)	—	0.01 (0.21)	0.04 (0.90)
CGM receiver	4.05 (0.58)	0.01 (0.21)	—	0.05 (0.90)
MyFitnessPal	4.10 (0.68)	0.04 (0.90)	0.05 (0.90)	—
Accelerometer	3.73 (0.76)	0.33 (0.55)^c^	0.32 (0.55)^c^	0.37 (0.98)^c^

^a^The Likert scale used in the ratings was 1=strongly disagree, 2=disagree, 3=neither agree nor disagree, 4=agree, 5=strongly agree.

^b^Continuous glucose monitor.

^c^*P*<.05 based on a two-tailed paired-sample *t* test.

Table 3 shows the mean summary acceptability score for each data collection tool. The summary acceptability score was comparable between the CGM sensor and MyFitnessPal (mean difference=–0.04, *P*=.79), and the CGM receiver and MyFitnessPal (mean difference=–0.05, *P*=.76). The summary score was approximately 4 for these data collection tools, suggesting participants overall “agreed” with the different aspects of acceptability for CGM sensor, CGM receiver, and MyFitnessPal. The accelerometer had a summary score of mean 3.73 (SD 0.76), which was significantly lower compared to the CGM sensor (*P*=.003), CGM receiver (*P*=.003), and MyFitnessPal (*P*=.048). This suggests that participants’ overall perception of the acceptability for accelerometer was between neutral to “agreed.”

## Discussion

### Principal Findings

Results from this study suggest high acceptability of using a CGM in a sample of free-living, nondiabetic adults. The overall acceptability for a CGM sensor and receiver was comparable to the diet tracking app MyFitnessPal and was higher than the waist-worn accelerometer. Participants recorded 5 to 6 eating events per day on average using the CGM receiver and MyFitnessPal, which is similar to other studies that used digital tools to collect dietary data [30]. These data suggest that participants were using the two devices as instructed during the monitoring period. After wearing the CGM sensor and using the CGM receiver for 1 week, more than 90% of the study participants agreed that the CGM sensor and receiver were easy to use, useful, and provided relevant information that was of interest to them. These results demonstrate a great potential for using CGM in nondiabetic adults as previous research has suggested that individuals will not engage with technology that is challenging to use or is perceived as irrelevant to their needs [28,31].

Percent agreement was low for the statement regarding feeling motivated to use the CGM sensor (63%) and receiver (67%). However, a post hoc analysis showed that these scores were comparable to the one for MyFitnessPal (80%; *P*=.20 and *P*=.29, respectively) and significantly higher than the one for the accelerometer (37%; *P*=.009 and *P*=.005, respectively). Potentially contributing to this finding is that both the CGM and the MyFitnessPal app provide feedback to the user regarding their glucose dynamics and their dietary intake, respectively, whereas the accelerometer does not. Feedback that is person-specific, actionable, and goal related tends to improve outcomes in interventions [32]. Furthermore, for Project SENSE, no specific explanations were given to participants about how their glucose pattern might reflect their behaviors (eg, dietary intake or physical activity). Therefore, this lack of knowledge of how data from CGMs might be related to their behaviors might have contributed to a low motivation score. To increase motivation, future studies considering the use of CGMs might want to provide a few examples of the potential effects of dietary intake and physical activity on glucose levels (eg, glucose will rise sharply after consuming high carbohydrate food; the more you move the more glucose you burn).

The CGM receiver also had lower agreement for the statement regarding convenience (63%). It is worth noting that the CGM model used in this study (Dexcom G4) required the receiver to be within 18 feet of the sensor at all times to ensure proper data transmission. Therefore, participants might have found it burdensome to always carry an additional device with them. Nevertheless, the need to have a receiver is being phased out in newer CGM models. For example, Dexcom G5 and G6 users can have the option to download a mobile app and use their mobile phone to receive and view glucose data instead of the receiver. For FreeStyle Libre CGM users (Abbott, Alameda, CA, USA), a receiver (ie, reader) is only needed at the time of retrieving glucose data (through scanning the reader over the sensor). Hence, as the technology for CGM system keeps advancing, it is expected that the concern regarding convenience will be minimized.

The overall acceptability score was similar for the CGM system and MyFitnessPal, but lower for the accelerometer. Other than the reasons discussed previously, one potential factor that might explain the differences in acceptability could be the different behaviors that were tracked by these tools. It is possible that individuals in this study perceived a food tracking tool (eg, MyFitnessPal) as more acceptable than a physical activity tracking tool (eg, accelerometer).

Although the CGM system could be regarded as an acceptable data collection tool in research settings, its usage beyond assessment purposes might be limited in the nondiabetic population if being used as is. Only 40% of the assessed participants from this study agreed that they would use the CGM, in its current form, for health and wellness purposes. As discussed previously, one of the reasons for this low endorsement for personal use of a CGM could be the lack of knowledge and ability to associate glucose number with health and wellness goals in this population. Individuals might need specific education and guidance to help them understand how their daily behaviors might impact their glucose pattern and subsequently influence their health. Future behavioral interventions exploring the use of CGMs in the nondiabetic population for health promotion purposes could provide appropriate information sessions and develop meaningful and actionable feedback messages to fully utilize the rich data from the CGM.

### Limitations

Although this study was among the few that used a CGM in a sample of nondiabetic adults and was the only one that assessed participant acceptability, it had some limitations. First, the study was not powered to formally test any difference in acceptability across different study tools (ie, CGM, mobile app, and accelerometer). Second, the majority of the study participants were highly educated and female. Therefore, findings from this study might not be generalizable to males or individuals with lower socioeconomic status. Further, participants in this study could be highly motivated because they volunteered to take part in the study. One of the possible reasons that individuals learned about the study but did not sign up could be they were not willing to wear the CGM. Therefore, this potential selection bias might contribute to the high acceptability of CGM in the study sample. Third, findings regarding the accelerometer might be limited to the specific model that was used in this study, which was a waist-worn passive device (ActiGraph GT3X) that participants were not able to interact with. This limitation in the user interface of the accelerometer could have greatly impacted participants’ rating in value, relevance, and motivation. Devices such as the ActiGraph GT3X have been considered “research grade” and thus remain one of the popular tools to monitor activity. However, as the technology of wearable activity monitors keeps improving, researchers will have more options when choosing assessment and intervention tools and should consider how different characteristics of the device (eg, placement location, integration with mobile phones) might affect users’ experiences. As an example, consumer devices such as Fitbit have been increasingly used in research [33,34] and have been shown to accurately quantify energy expenditure and steps taken [35-39]. Similarly, findings regarding CGMs might be limited to the specific type of technology (eg, the requirement of finger prick calibration, insertion site of the sensor). For example, the finger prick requirement could be perceived as a barrier for CGM use. Yet, the CGM system scored higher on overall acceptability compared to the accelerometer. It is possible that the novelty of the CGM and the ability to see their glucose number outweighed the temporary burden of finger pricks in this study population. Whereas participants could have viewed the accelerometer as more encumbered because they had to wear it on their body without getting any information out of it. It is worth noting that the newer generation of CGMs have eliminated the need for finger prick calibration (ie, the Dexcom G6 and Freestyle Libre). As the CGM technology keeps improving (ie, becomes less invasive), the acceptability of its use in the nondiabetic population could potentially increase. This is evidenced by feedback from this study showing that 80% would use the CGM for health promotion purposes if it was less invasive. Another potential difference in CGM models that could impact acceptability is the insertion site of the sensor. For example, Dexcom devices are inserted on the abdomen, whereas the Freestyle Libre devices are inserted on the back of the upper arm. Some individuals might perceive the abdomen area as a more hidden placement site than the back of the upper arm, although other individuals might have the opposite preference. Nevertheless, this difference in insertion sites might have a greater impact for individuals that need to wear the device long term (ie, diabetic patients) compared to individuals that only need to wear the device short term such as for research purposes. Further, findings from this study could be limited by the design in which participants were required to log meals and physical activity using both the CGM receiver and MyFitnessPal. This could have potentially increased participant fatigue and affected their acceptability ratings. Often the reason for using multiple data collection tools simultaneously in a research setting is to obtain information about participant behaviors and the subsequent impact that is as complete as possible, with each tool will providing different kinds of data (eg, glucose response from CGM and calories consumed from MyFitnessPal). Therefore, participants will be more likely to be required to use multiple devices for research purposes. Nevertheless, it should be kept in mind that participants could have different feelings toward the tools if each of them was used disjointedly. Lastly, this study assessed feasibility using a purely quantitative approach. Although this approach allowed a more straightforward comparison between tools and is easier to administer compared to a qualitative approach, a qualitative approach using interviews or focus groups would have offered some more in-depth information about acceptability from the participants. Nevertheless, this study did include an open-ended question asking participants what they liked most about this study. The most frequently mentioned aspect was “being able to see the glucose number in real time” (mentioned by 73% of the participants) followed by “monitoring food intake using MyFitnessPal” (mentioned by 60% of the participants).

### Implications and Conclusions

The continued advancement in technology will further diversify the use of wearable devices and foster innovative approaches in diet and physical activity research. The CGM represents one type of biological sensor that has the potential to provide personalized physiological data for a biomarker that is closely related to dietary and physical activity behaviors. These data could potentially be used to present the immediate or short-term (eg, past 24 hours) physiological consequences of dietary and physical activity behaviors as a strategy to encourage positive changes in those behaviors [21]. The ability to frequently assess a marker related to a behavioral goal is the foundation for providing just-in-time feedback that could ultimately optimize strategies for impactful behavioral changes [32].

Results from this study suggest that although healthy individuals do not mind wearing a minimally invasive CGM device for 1 week, the motivation for wearing it was moderate, possibly due to the lack of ability to interpret or make sense of all the data that were available to them. As the barriers to tracking and collecting health behavior data are overcome by technological advancements, the challenge ahead will be determining how to most efficiently and effectively use these data to provide meaningful insights and useful feedback to users. Thus, more behavioral research that uses CGMs and other biological sensors is needed to offer evidence-based recommendations that assist individuals with their behavior change goals.

## Abbreviations

CGM: continuous glucose monitor
